# Supplementary material for: Patterns of Intron Gain and Loss in Fungi
Source: PLoS Biol. 2004 Nov 30;2(12):e422. doi: 10.1371/journal.pbio.0020422 (PMC532390; doi:10.1371/journal.pbio.0020422)
Supplement: Table S1 — Also available at http://genes.mit.edu/NielsenEtAl/. (4.3 MB ZIP). [file pbio.0020422.st001.zip › NielsenEtAl/html/197.html]

AN8848.1.NCU06198.1.MG08858.1.FG02699.1


```
 CLUSTAL W (1.82) Multiple Sequence Alignments - Introns Inserted


Sequence 1: NCU06198.1	392 aa
Sequence 2: MG08858.1	394 aa
Sequence 3: FG02699.1	390 aa
Sequence 4: AN8848.1	428 aa
Alignment Length: 434 aa
Number Identitical Residues: 182 aa
Alignment Score (without introns) 8840


MG08858.1 	MAD--KKNEDFV~VRMPDN--------GTVEKEPFLAR----------------------
NCU06198.1	MAN--KRNEDIE~LGPAEGR-------GSTDKDPFLARR---------------------
FG02699.1 	MADQNKKNDDFV~AKMPEVANENNIGDGEKENDSLVGR----------------------
AN8848.1  	MVEGRKGKLCEV1LESPSFFGTLPVFNRYLFAKSLLYRPNSLIHHAATVIMTDNRKPEDY
          	*.:  * :        ..  .     .     ..:: * .:    :::   :.. .... 

MG08858.1 	-------SPPARARTGSGGGFGDSFSLARVANNPPAAILAYCLSSISMTVVNKYVVSGSE
NCU06198.1	-------SSSQPNRPQQAGPFGGYF--DKIDHSPGASIIAYCLSSISMTVVNKYVVSGSE
FG02699.1 	-------GPGTDALPAQPGGF-----MATVENSGPFSILAYCLSSISMTVVNKYVVSGSS
AN8848.1  	TIEMDKLGQNKNYQAPPPPPQPRSSTASSISNNAALSVLAYCGSSILMTVMNKYVLS-SD
          	: . .. .      .          :   : :.   :::*** *** ***:****:* *.

MG08858.1 	WNLNFFYLAVQ0AIVCIIAILFCKQIGIITNLAPFDNVKAKKW1FPVSLLLVGMIYTSTK
NCU06198.1	WNLNFFYLAVQ0SLVCTAAILICKQLGMFQNLAAFDSTKAKKW1FPISLLLVGMIYTSTK
FG02699.1 	WNLTFFYLAVQ0SIVCIAAITACKQLGMIKSLAPLEMDRIKKW1YPISLVLVGMIYTSTK
AN8848.1  	FNLNFFLLCVQ0SLVCIIAIQLCKACGLIT-YRDFNLDEARKW1FPITLLLIGMIYTGSK
          	:**.** *.** ::**  **  **  *::     ::  . :** :*::*:*:*****.:*

MG08858.1 	ALQFLSVPVYTIFKNLTIIAIAYGEVLWFGGSVSPLALVSFGLMVLSSVVAAWADIQSAI
NCU06198.1	ALQFLSVPVYTIFKNLTIIVVAYGEVLWFGGSVTPMALLSFGLMVLSSVIAAWADIQAAV
FG02699.1 	ALQYLSVPVYTIFKNLTIIAIAYGEVLWFGGSVTSTALASFSLMVLSSVVAAWADIKSAI
AN8848.1  	ALQFLSIPVYTIFKNLTIILIAYGEVLWFGGSVTNLTLFSFGLMVFSSIIAAWADIKHAI
          	***:**:************ :************:  :* **.***:**::******: *:

MG08858.1 	HG-GSHPSEASTAISTLNAGYAWMGMNVFCSAAYLLGMRKVIHKMNFKDWDS1MFYNNLL
NCU06198.1	EG-VGHTAEATDAISTLNAGYAWMGMNVFCTAAYLLGMRKVIKKMNFKDYDT1MFYNNLL
FG02699.1 	SGDYSATTGDADALATLNAGYFWMAMNVICSASYVLGMRKVIHKMNFKDWDT~MYYNNLL
AN8848.1  	ES----SGDATSKVSTLNAGYIWMLINCLCTSSYVLGMRKRIKLTNFKDFDT1MFYNNLL
          	 .    .   :  ::****** ** :* :*:::*:***** *:  ****:*: *:*****

MG08858.1 	TIPVLIVCSLIAEDWSAANLARNFPIESRNALFIGMIYSGLGAIFISYCSAWCIRVTTST
NCU06198.1	TIPVLIVFSLLFEDWSNDNLIKNFPVETRNSLFIGMIYSGLAAIFISYCSAWCIRVTSST
FG02699.1 	TIPVLVFFSLVTEDWSSANLAKNFPEDSRNRMMIGIIYSGLAAIFISYCSAWCIRVTSST
AN8848.1  	SIPVLIVCSGILEDWSPANVARNFPSADRNGIMFAMILSGLSTVFISYTSAWCVRVTSST
          	:****:. * : ****  *: :***   ** :::.:* ***.::**** ****:***:**

MG08858.1 	TYSMVGALNKLPIAISGLVFFSAPVTFGSVSAIVIGFISGIVYAWAKARQSSQAKSALPT
NCU06198.1	TYSMVGALNKLPLAISGLIFFDAPVTFGSVTAIFVGFVSGLVYTWSKTRQ--KVSQILPT
FG02699.1 	TYSMVGALNKLPIAVSGLIFFAAPVTFGSVSAIFIGFVSGIVYAWAKVKENEAKKNALPT
AN8848.1  	TYSMVGALNKLPIALSGLIFFDAPVTFPSVSAIMVGFVSGIVYAVAKIKQNAKPKVGILP
          	************:*:***:** ***** **:**.:**:**:**: :* ::.   .  : .

MG08858.1 	QQPVMSASSQSNKDASNS
NCU06198.1	TQPTMSASAASNRDAANA
FG02699.1 	AE---------NRDSSK-
AN8848.1  	TTNPVSASSQSMRDSLRS
          	     :::: : :*: .:
```
